# Efficiency of micro-nebulized hydrogen peroxide dry mist in improving disinfection of titanium and polypropylene surfaces used in the medical area

**DOI:** 10.1186/2047-2994-4-S1-P44

**Published:** 2015-06-16

**Authors:** ML Cavalleri, L Brambilla, C Meroni, P Rinaldoni

**Affiliations:** 1 Microbiology and Virology, Eurofins Biolab Srl GLP Test Facility, Vimodrone, Italy

## Introduction

Most hard surface cleaning and disinfection procedures adopted in healthcare facilities are not effective in inactivating bacteria where a biofilm is already formed and may be a cause of cross-contamination of clean areas. Airborne disinfection performed with micro-nebulized H2O2 dry particles may be used as additional step capable to inactivate the bacteria and prevent clean areas cross-contamination.

## Objectives

Objective of this study was to determine the efficiency of micro-nebulized hydrogen peroxide (H2O2) based dry mist in the disinfection of metallic and polymeric surfaces contaminated with a mature *Pseudomonas aeruginosa* biofilm.

## Methods

The biofilm of was prepared according to modified ISO/TS 15883-5 Annex F. The surface cleaning and disinfection was performed according to the authorized label of the disinfectant (wiping). The wiping test was performed following CEN quantitative carrier test method with mechanical action (4-field test) where one field was contaminated with the biofilm and the other three fields left clean. The test surface was wiped with microfiber wipes impregnated with 16 ml of disinfectant starting from the contaminated area and covering one by one all the other three clean areas. The airborne efficacy tests were performed in a climatized room of 50 cubic meters with the metal and polymeric contaminated carriers placed 2.6 meters away from the micro-nebulizer.

## Results

Micro-nebulized H2O2 particles showed efficacy against the biofilm if this was preliminarily treated with the detergent disinfectant. The micro-nebulization treatment caused a reduction of almost 3Logs of cfu/25cm^2^ in field one (after 20 minutes dry mist erogation and 3 hours exposure) whereas the preliminary detergent disinfection process showed a negligible reduction in field one. The cross-contamination of the clean fields which occurred during the wiping step was inactivated by the airborne disinfection step.

## Conclusion

Micro-nebulized H2O2 dry mist is capable of inactivating bacterial mature biofilm if the biofilm is pre-treated with a detergent, regardless of the type of material inoculated. H2O2 airborne disinfection reduces cross-contamination of clean areas also preventing new biofilm formation.

## Disclosure of interest

None declared.
